# Alcohol-induced plasticity in the dynorphin/kappa-opioid receptor system

**DOI:** 10.3389/fnmol.2012.00095

**Published:** 2012-09-27

**Authors:** Sunil Sirohi, Georgy Bakalkin, Brendan M. Walker

**Affiliations:** ^1^Laboratory of Alcoholism and Addictions Neuroscience, Department of Psychology, Washington State UniversityPullman, WA, USA; ^2^Division of Biological Research on Drug Dependence, Department of Pharmaceutical Biosciences, Uppsala UniversityUppsala, Sweden; ^3^Translational Addictions Research Center, Washington State UniversityPullman, WA, USA; ^4^Graduate Program in Neuroscience, Washington State UniversityPullman, WA, USA

**Keywords:** alcohol, dependence, depression, dynorphin, ethanol, kappa-opioid receptor, negative affect, withdrawal

## Abstract

Alcoholism is a chronic relapsing disorder characterized by continued alcohol use despite numerous adverse consequences. Alcohol has been shown to interact with numerous neurotransmitter systems to exert its pharmacological effects. The endogenous opioid system (EOS) has been strongly implicated in the positive and negative reinforcing effects of alcohol. Traditionally recognized as dysphoric/anhedonic in nature, the dynorphin/kappa-opioid receptor (DYN/KOR) system has recently received considerable attention due to evidence suggesting that an upregulated DYN/KOR system may be a critical contributor to the complex factors that result in escalated alcohol consumption once dependent. The present review will discuss alcohol-induced plasticity in the DYN/KOR system and how these neuroadaptations could contribute to excessive alcohol seeking and consumption.

## Introduction

Alcohol use disorders, comprising alcohol abuse and dependence, pose a substantial physical, mental, and fiscal health risk to millions of people each year in the US, causing an average of 79,000 deaths and costing $224 billion annually (Grant et al., 2004; Bouchery et al., 2011). Alcohol exerts it's effects through multiple neurotransmitter systems, e.g., dopamine (DA), glutamate, γ-aminobutyric acid (GABA), and serotonin (5-HT) systems (Colombo et al., 2004; Johnson, 2004; Walker and Ettenberg, 2007; Heinz et al., 2009). In particular, the endogenous opioid system (EOS) has proven to be important when considering the positive reinforcing effects of alcohol. Furthermore, the EOS undergoes neuroadaptations following chronic alcohol exposure that lay the foundation for alcohol to serve as a potent negative reinforcer during both acute and protracted withdrawal following chronic alcohol exposure (for a review of negative reinforcement from a learning perspective, see Walker, 2012).

Acute alcohol stimulates the release of β-endorphin (βEND), enkephalin (ENK), and dynorphin (DYN) (Gianoulakis et al., 1996; Marinelli et al., 2003, 2004, 2005, 2006; Dai et al., 2005; Lam et al., 2008; Jarjour et al., 2009). βEND and ENK, endogenous ligands for μ-(MOR) and δ-(DOR) opioid receptors, respectively, have been linked to euphoric and positive reinforcing effects of alcohol (Stromberg et al., 1998; Hyytia and Kiianmaa, 2001). Conversely, DYN, the endogenous ligand for the κ-opioid receptors (KORs) (Chavkin et al., 1982), has been shown to produce aversive effects related to alcohol challenge (Lindholm et al., 2001). There are two forms of DYN, DYN A, and DYN B, although for the purposes of this review, they will be collectively called DYN because the precise neurobehavioral differences between the two has yet to be established. The KOR is the preferential binding site for DYN (Chavkin et al., 1982; Merg et al., 2006), although DYN has affinity for all three opioid receptors (Merg et al., 2006; Schwarzer, 2009).

The KOR, a G-protein coupled receptor, induces inhibitory signaling (Connor and Christie, 1999; Al-Hasani and Bruchas, 2011; but see Crain and Shen, 1990) and has been shown to regulate the release of various neurotransmitters including DA, glutamate, GABA, norepinephrine (NE), and 5-HT (Mulder et al., 1984; Jackisch et al., 1986; Schoffelmeer et al., 1997; Shippenberg and Rea, 1997; Rawls et al., 1999; Shippenberg et al., 2007; Land et al., 2009). Details of these interactions have been reviewed previously (Vengeliene et al., 2008; Heinz et al., 2009). The dynorphin/kappa-opioid receptor (DYN/KOR) system is widely distributed in the CNS and has been implicated in numerous physiological and pathophysiological conditions related to mood and motivation (Bruijnzeel, 2009; Schwarzer, 2009; Wee and Koob, 2010; Tejeda et al., 2012), identifying the DYN/KOR system as a putative therapeutic target for the treatment of various neuropsychiatric disorders (Walker and Koob, 2008; Knoll and Carlezon, 2010; Wee and Koob, 2010; Tejeda et al., 2012; Walker et al., 2012). Also becoming apparent, is the importance of the dysphoric/anhedonic properties of a hyperactive DYN/KOR system in alcohol dependence that contributes to the negative reinforcing effects of alcohol (Walker and Koob, 2008; Nealey et al., 2011; Walker et al., 2011). The present review focuses on alcohol-induced plasticity in the DYN/KOR system and how these neuroadaptations perpetuate excessive alcohol seeking and consumption.

## The DYN/KOR system in motivational and emotional neurocircuitry

**Figure 1:**
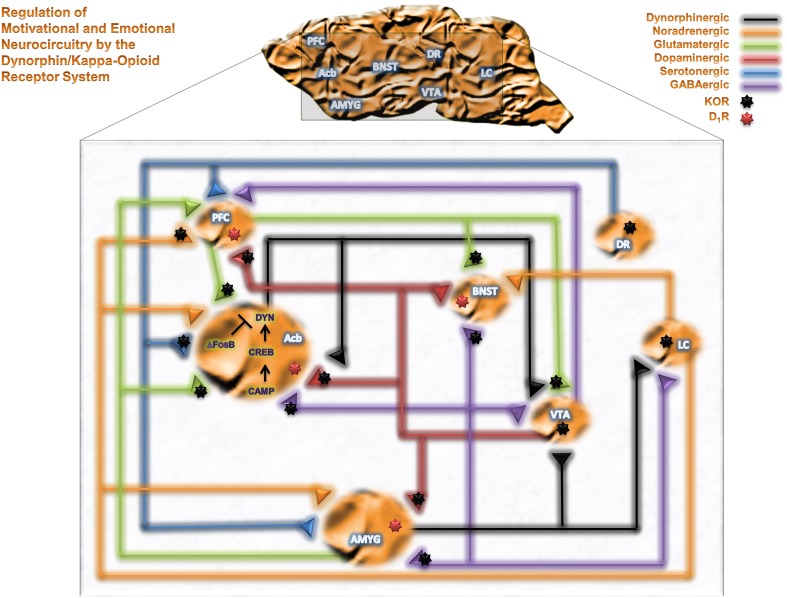
**The DYN/KOR system in motivational and emotional neurocircuitry.** A schematic representation of brain motivational and emotional neurocircuitry, illustrating relevant neurotransmitters implicated in the pathophysiology of various neuropsychiatric disorders following chronic exposure of alcohol and drugs of abuse. Abbreviations: PFC, prefrontal cortex; Acb, nucleus accumbens; AMYG, amygdala; BNST, bed nucleus of the stria terminalis; VTA, ventral tegmental area; DR, dorsal raphe; LC, Locus coeruleus; GABA, γ-aminobutyric acid; DYN, dynorphin; cAMP, cyclic AMP, CREB, cAMP response element-binding protein; ΔFosB, a member of the Fos family of transcription factors.

Neuropharmacological studies have identified brain regions mediating the reinforcing effects of alcohol and other drugs of abuse. The mesolimbocortical dopamine system [DA from the ventral tegmental area (VTA) to the nucleus accumbens (Acb) or prefrontal cortex (PFC)] and extended amygdala [central nucleus of the amygdala (CeA), bed nucleus of the stria terminalis (BNST) and Acb Shell (AcbSh)] represent neurocircuitry related to motivational and cognitive decision making, as well as emotional, neurocircuitry thought to participate in the reinforcing effects of alcohol. Figure 1 summarizes brain motivational and emotional neurocircuitry and illustrates several relevant neurotransmitter systems that can be modulated by KORs and are implicated in the pathophysiology of various psychiatric disorders following exposure to drugs of abuse, including alcohol. KORs are presynaptically positioned on dopaminergic inputs to amygdala (Amyg), Acb and PFC (Werling et al., 1988; Frankhuijzen et al., 1991; Grilli et al., 2009), GABAergic inputs to Acb, Amyg and BNST (Hjelmstad and Fields, 2003), serotonergic inputs to Acb (Land et al., 2009), noradrenergic inputs to PFC (Berger et al., 2006) and glutamatergic inputs to VTA and Acb (Hjelmstad and Fields, 2001). KORs are also found directly on the perikarya of dorsal raphe 5-HT neurons (Land et al., 2009), locus coeruleus NE neurons (Reyes et al., 2009) and VTA mesocortical DA neurons (Margolis et al., 2006). As such, KORs are positioned to modulate numerous neurotransmitter systems implicated in neuropsychiatric disorders within motivational and emotional circuitry (Heinz et al., 2009; Schwarzer, 2009).

## Alcohol-induced plasticity in the DYN/KOR system

**Figure 2:**
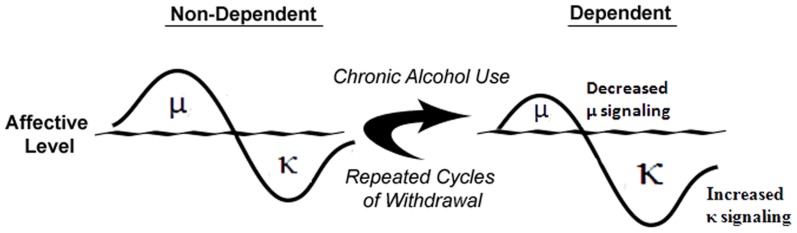
**Alcohol dependence-induced change in opioidergic system.** In non-dependent organisms, alcohol-induced positive affective states mediated by the μ-opioid receptor precede compensatory negative affective states expressed through the κ-opioid receptor. Following chronic alcohol exposure, μ-opioid receptor signaling is attenuated and through multiple mechanisms, κ-opioid receptor signaling is increased to produce increased negative affective states. Adapted from Walker et al. (2012).

Understanding the acute neurobiological effects of alcohol is critically important because once known, it may be possible to predict the neuroadaptive, and resulting, behavioral impact of long-term alcohol exposure using theories such as the opponent-process theory of motivation (OPT; Solomon and Corbit, 1974). If applying this theory to alcoholism, in order to maintain homeostasis, an increase in hedonic state (e.g., alcohol-induced euphoria) will be followed by a compensatory decrease in hedonic state (Figure 2). Furthermore, after repeated alcohol exposure, the positive hedonic state is reduced while the negative component is enhanced to compensate for the continued perturbation of the affective system by chronic alcohol exposure (Figure 2). As the cycle continues, the cessation of alcohol intake would result in the production of a negative affective state during withdrawal that can drive an organism to excessively seek and use alcohol. In accordance with the OPT, if alcohol-mediated MOR or DOR stimulation (Marinelli et al., 2004, 2005; Lam et al., 2008; Jarjour et al., 2009) produces positive hedonic states (Amalric et al., 1987; Shippenberg et al., 1987), then a compensatory mechanism could be increased DYN and/or function of KORs, stimulation of which produces negative hedonic states (Mucha and Herz, 1985). Under conditions of chronic alcohol use (see Figure 2), the predicted response of the endogenous opioidergic system would be attenuated MOR signaling and exacerbated DYN/KOR system activity, both of which are supported in the literature (Gianoulakis et al., 1996; Przewlocka et al., 1997; Turchan et al., 1999; Chen and Lawrence, 2000; Lindholm et al., 2000; Saland et al., 2004; Lindholm et al., 2007). In addition, chronic alcohol exposure has been shown to alter various neuropeptide systems (e.g., corticotropin-releasing factor (CRF), neuropeptide Y and nociceptin; Cowen and Lawrence, 2006; Ciccocioppo et al., 2009; Koob, 2010) that may contribute to the development of alcohol dependence and/or negative affective states. Therefore, a “counter-regulatory” the DYN/KOR system is recruited following exposure to alcohol and other drugs of abuse. Repeated alcohol exposure upregulates the DYN/KOR system and creates a state that facilitates alcohol seeking and consumption. The precise mechanisms that underlie escalated alcohol consumption in alcohol dependent states may involve adaptations at the pharmacological, transcriptional, and epigenetic levels and are discussed below.

### Pharmacological evidence

It has been proposed that the neuroadaptive changes that occur in response to chronic alcohol use can occur via within- or between-system changes in reward and anti-reward systems, respectively (Koob and Bloom, 1988; Koob and Le, 2008; Koob, 2009). There is evidence supporting both possibilities in the form of neuroadaptations that occur within classical motivational systems (Koob and Weiss, 1992; McBride and Li, 1998; Siggins et al., 2003; Koob, 2004; Funk and Dohrman, 2007), as well as systems distinct from those that are involved in anhedonia and dysphoria (Valdez et al., 2002; Funk et al., 2006; Walker and Koob, 2008; Sperling et al., 2010; Nealey et al., 2011; Walker et al., 2011). Although DA and the EOS within the mesolimbic pathway, ventral striatum and CeA participate in dependence-induced within-system changes and stress-related peptides in the extended amygdala are hypothesized to participate in between-system changes [see the excellent review, (Koob, 2009)], there are several other neuropeptides involved in the positive and negative reinforcing effects of alcohol (Cowen and Lawrence, 2006; Koob and Le, 2008; Ciccocioppo et al., 2009; Gilpin and Roberto, 2012) that show great promise as therapeutics for the treatment of certain aspects of addictive disorders. Strong support for the OPT is recent evidence demonstrating that acute alcohol administration initially increases β END within the first 30 min that is followed by a significant increase in DYN A approximately 1.5–2 h later (Lam et al., 2008; Jarjour et al., 2009); a profile that is also observed within the Acb and VTA (Marinelli et al., 2004, 2006; Jarjour et al., 2009). The OPT predicts that chronic alcohol exposure would decrease positive affect and increase negative affect (see Figure 2). In support of that prediction, evidence has shown that the MOR– and DOR–regulated component of the opioid system shows decreased signaling in response to chronic alcohol (Turchan et al., 1999; Chen and Lawrence, 2000; Saland et al., 2004). Also consistent with that hypothesis, chronic alcohol–exposed animals have been shown to have increased prodynorphin mRNA levels in the Acb (Przewlocka et al., 1997), increased expression of DYN B in the Acb (Lindholm et al., 2000) and altered KOR mRNA expression in the Acb and VTA (Rosin et al., 1999) that support the concept of an upregulated DYN/KOR system in these areas.

The functional impact of increased DYN/KOR system activity in dependence involves, in part, the mesolimbocortical DA system. This system has been implicated as a signaling system for biologically relevant information through which drugs of abuse (Koob, 2000; Maldonado, 2003; Di et al., 2004) and natural reinforcers (Hull et al., 1999; Carelli, 2002; Kelley et al., 2002, 2005) or punishers can exert their behavioral effects due to the mesolimbic DA pathway's capacity for bidirectional signaling (i.e., ability to signal both positive and negative stimuli; Wheeler and Carelli, 2009). Within the mesolimbic DA system, KORs are neuroanatomically positioned on DA terminals in the AcbSh that enables them to oppose the effects of MOR agonists on DA release (Di and Imperato, 1988). KORs are also positioned on DA perikarya in the VTA (Margolis et al., 2003, 2006). Much research has been done to determine how KOR stimulation impacts dopaminergic neurotransmission and drug self-administration, (for an excellent review, see Shippenberg et al., 2007). In essence, while the KORs positioned on the terminal regions in the AcbSh reduce DA release (Di and Imperato, 1988) in that region, KORs on VTA DA neurons do not (Spanagel et al., 1992; Margolis et al., 2006), but instead selectively reduce DA release in the PFC (Margolis et al., 2006). Thus, increased signaling through DYN/KOR system could functionally result in an attenuated dopaminergic system in both cortical and limbic circuitry. Indeed, deficiencies in dopaminergic transmission have been posited by some to be the neurobiological basis of depression (Nestler and Carlezon, Jr., 2006).

Substantial evidence supports the concept of chronic alcohol–induced attenuation of DA (Carroll et al., 2006; Healey et al., 2008). Stimulation of KORs produces dysphoria in humans (Pfeiffer et al., 1986) and place aversions in animals (Mucha and Herz, 1985). Furthermore, increased DYN transmission in the Acb has been hypothesized to induce depressive–like behavioral states in animal models of depression and negative affect (Pliakas et al., 2001; Mague et al., 2003; Carlezon, Jr. et al., 2006). The DYN/KOR system in the basolateral amygdala (BLA) has been implicated as a mediator of dysphoria through which stress–related systems can exert their effects (Land et al., 2008). Thus, if a compensatory response to chronic alcohol involved alterations in DYN/KOR signaling, then DYN/KOR–mediated negative affect could contribute to the increased alcohol consumption observed in dependence. Taken together, increased DYN transmission could result in attenuated dopaminergic transmission and produce depressive–like behaviors and dysphoria that are thought to involve multiple nuclei within extended Amyg circuitry. Therefore, if the DYN/KOR system is upregulated following chronic alcohol exposure in a manner sufficient to produce escalated alcohol self-administration (Roberts et al., 2000; O'Dell et al., 2004; Walker and Koob, 2008), KOR antagonists should be able to reduce negative affective states associated with withdrawal and reduce the excessive alcohol self-administration. Recent studies substantiating this hypothesis demonstrated that systemic, intracerebroventricular and intra-AcbSh administration of a KOR antagonist were able to selectively reduce escalated operant self-administration of alcohol in dependent Wistar rats while leaving nondependent alcohol self-administration intact (Walker and Koob, 2008; Nealey et al., 2011; Walker et al., 2011). These selective effects of KOR antagonists in dependent animals strongly implicate the recruitment of DYN/KOR system during the transition to alcohol dependence.

Although considerable work has focused on KOR modulation of DA transmission, KORs within the extended amygdala can presynaptically modulate other neurotransmitters (e.g., glutamate, GABA and serotonin; Fields et al., 2007; Land et al., 2009; Li et al., 2012). As many of these neurotransmitter systems have been implicated in alcohol reinforcement and dependence, upregulated DYN/KOR activity could be a common mechanism that adversely impacts motivational and emotional neurocircuitry. Specifically, KORs can presynaptically inhibit GABAergic signaling in the BNST that is thought to remove an important inhibitory influence on glutamatergic neurons and could contribute to the hyperglutamatergic state observed during alcohol withdrawal (Spanagel, 2009; Li et al., 2012). Furthermore, serotonergic projections from the dorsal raphe to limbic brain regions (Hensler, 2006) that can regulate affect and drug seeking behavior (Land et al., 2009; Bruchas et al., 2011) are impacted by alcohol exposure (Chu and Keenan, 1987; Pistis et al., 1997) and may be dysregulated during alcohol dependence (Gorwood et al., 2000; Shibasaki et al., 2010). KORs can also impact 5-HT levels via modification of efflux/tone (Tao and Auerbach, 2005) and appear to mediate aversive behavior in mice (Land et al., 2009). A recent study suggested that KOR mediated p38 MAPK signaling can induce serotonin transporter (SERT) translocation from the intracellular pool to the neuronal membrane, thereby enhancing serotonin reuptake (Land et al., 2009; Bruchas et al., 2011) and causing a hyposerotonergic state. Furthermore, Shibasaki and colleagues have shown upregulated SERT mRNA in the dorsal raphe of alcohol-dependent rodents (Shibasaki et al., 2010). Taken together, DYN/KORs in this circuitry may be upregulated in alcohol dependence and may reduce serotonergic tone through a p38 MAPK-dependent SERT translocation mechanism that can contribute to the development of a negative affective state. Modulation of this state has been shown to manage certain symptoms of rapid alcohol exposure (Johnson, 2004; Uzbay, 2008), although the face and predictive validity of the models used to evaluate alcohol withdrawal should be carefully scrutinized. Another mechanism through which KORs can regulate affect was identified by Hjelmstad and Fields (2001) who documented that KORs are also anatomically positioned on presynaptic terminals of glutamatergic inputs from the PFC, Amyg and hippocampus to the Acb. Therefore, in addition to local blockade of DA release in the Acb by DYN/KOR, KOR modulation of glutamatergic inputs to the Acb may also be involved with aversive behaviors; however, additional studies are needed to confirm this. These studies may help to explain the significant comorbidity between alcohol use disorders and affective disorders (Regier et al., 1990; Grant and Harford, 1995) that can plague those afflicted with alcohol dependence.

The EOS may also have a role in certain cognitive processes relevant for control of addictive behavior including craving, decision-making and impulsivity (O'Malley et al., 2002; Bencherif et al., 2004; Boettiger et al., 2009; Love et al., 2009). Thus, a dysregulated EOS may contribute to impaired neurocognitive function and reduced regulation of alcohol/drug seeking and consumption. A recent study demonstrated a significant increase in PDYN mRNA (PDYN is the precursor peptide for the two forms of DYN, DYN A, and DYN B) in the dorsolateral prefrontal cortex (dl-PFC), and KOR mRNA in the orbitofrontal cortex (OFC) of deceased alcoholics when compared to controls (Bazov et al., 2011; Taqi et al., 2011b). Furthermore, levels of both DYN A and DYN B were significantly elevated in the dl-PFC and hippocampus of alcoholics. Importantly, the levels of PDYN mRNA significantly correlated with those of DYN peptides. These alterations were observed in brain regions involved in cognitive control of addictive behavior. Therefore, DYNs may have a role in regulation of executive functions and their elevation may impair these cognitive processes. In addition, DYN A can induce effects that are not blocked by opioid antagonists (Faden and Jacobs, 1983; Dubner and Ruda, 1992; Caudle and Mannes, 2000; Lai et al., 2001; Tan-No et al., 2001, 2005; Singh et al., 2003; Hauser et al., 2005). These non-canonical non-opioid effects, generally excitatory, may lead to neurodegeneration and pathological behavior such as chronic pain and paralysis. Therefore, both the opioid-receptor mediated and non-opioid neurodegenerative mechanisms may underlie the behavioral effects of upregulated DYN/KOR system in alcoholics.

### Transcriptional evidence

Following DA release in motivational nuclei (i.e., Acb), it binds to its receptors (D_1_- or D_2_-like) and produces excitatory or inhibitory post-synaptic potentials, respectively. D_1_-like receptor activation increases adenylyl cyclase activity by coupling to stimulatory G proteins (Gαs). This leads to an increase in the concentration of cyclic AMP (cAMP) and potentiation of cAMP-dependent protein kinases A (PKA) activity that further phosphorylates downstream signaling substrates. One signaling substrate is the transcription factor cAMP response element-binding protein (CREB) that activates the transcription of *PDYN*, *BDNF*, *CRF*, *NPY*, and other genes (Lonze and Ginty, 2002; Carlezon, Jr. et al., 2005). CREB-mediated increases in DYN within the Acb serves as a negative feedback circuit whereby it decreases DA release in Acb through presynaptic receptors on DA containing nerve terminals (see Figure 1). This is evident from experiments showing that KOR antagonist blocks the effects of CREB over-expression (Carlezon, Jr. et al., 1998). Because alcohol exposure may alter CREB mediated pathway in the Acb (Misra et al., 2001), mice lacking a regulatory subunit of PKA show attenuated cAMP-PKA signaling in the Acb and increased alcohol consumption (Thiele et al., 2000). In addition, PKA inhibition, that further decreases PKA/CREB activity, produces high alcohol preference in rodents (Misra and Pandey, 2006). Activation of CREB results in elevation of DYN levels in this circuitry (Carlezon, Jr. et al., 1998). Upregulated CREB signaling in the VTA-Acb pathway produces pro-depressive behavior (Pliakas et al., 2001; Malberg and Blendy, 2005), while CREB or DYN inhibition in the Acb produces an antidepressant-like effect (Newton et al., 2002). Early exposure to methylphenidate that causes sustained elevation in CREB in Acb produces anhedonia and dysphoria (Bolanos et al., 2003; Carlezon, Jr. et al., 2003). Collectively, CREB-mediated DYN upregulation in the Acb may mediate reduced reward and pro-depressive effects of chronic alcohol and drug exposure. In the Amyg, alterations in CREB-mediated signaling following alcohol exposure has been implicated in anxiety associated with alcohol withdrawal, albeit in a direction opposite of that in the Acb (Pandey et al., 2005); however, the time-points of CREB evaluation in the Amyg (although during acute withdrawal) were more protracted than in other investigations (24 h vs. 6–10 h into withdrawal; (Pandey et al., 2005; Williams et al., 2012, respectively). Thus, CREB-mediated increases in DYN within critical brain regions implicated in mood regulation may contribute to the development of negative affective behavior during withdrawal in dependent organisms.

The cAMP signaling pathway is one mechanism by which alcohol and other drugs of abuse can alter DYN concentrations in the Acb via CREB-mediated transcriptional activation. In addition to directly targeting the prodynorphin gene, CREB may regulate several signaling pathways (Carlezon, Jr. et al., 2005) that may activate the DYN/KOR system following chronic exposure to alcohol or addictive drugs (Carlezon, Jr. et al., 2005; Nestler and Carlezon, Jr., 2006). Brain- derived neurotropic factor (BDNF) is an important CREB target (Lonze and Ginty, 2002) that modulates DYN expression in brain region specific manner (Nair and Vaidya, 2006). BDNF, a member of the nerve growth factor (NGF) family, and its receptor TrkB are widely distributed throughout the brain (Wetmore et al., 1990; Altar et al., 1994), and have a role in synaptic plasticity (Chao, 2003) associated with alcohol addiction (Uhl et al., 2001) and several psychiatric disorders (Martinowich et al., 2007). BDNF expression is associated with early onset of alcoholism (Matsushita et al., 2004). Alcohol exposure increases BDNF expression in the dorsal striatum (DS) leading to prodynorphin activation (Logrip et al., 2008). These effects are brain region specific (Nair and Vaidya, 2006) and further work is needed to understand the precise pathophysiological mechanisms of dysregulated CREB-BDNF signaling in alcohol dependence. Thus, alcohol and other substances of abuse may dysregulate CREB-mediated signaling in the Acb leading to DYN upregulation. That upregulation appears to contribute to the depressive and aversive effects of alcohol, cocaine and other illicit drugs (Carlezon, Jr. et al., 1998, 2005; McClung and Nestler, 2008).

One transcription factor that gained attention for its putative role in long-lasting plastic changes underlying addiction is ΔFosB (Nestler et al., 2001). ΔFosB is a member of the Fos family of transcription factors encoded by the *FOS* genes. Fos proteins are rapidly, but for a short period of time, induced in Acb and dorsal striatum, brain regions involved in the rewarding and locomotor effects of various drugs of abuse and alcohol (Nye and Nestler, 1996; Kelz et al., 1999; Kelz and Nestler, 2000; Perrotti et al., 2008). Following chronic treatment with alcohol or drugs of abuse only ΔFosB has been found to accumulate in many brain regions related to goal-directed actions and decision-making, including the striatum and PFC (Hope et al., 1994; Kelz and Nestler, 2000; Perrotti et al., 2008). ΔFosB is an unusually stable, C-terminally truncated variant of the immediate early gene product FosB. ΔFosB is thought to function as a sustained molecular switch for addiction. Chronic exposure of rodents to most drugs of abuse, including cocaine, morphine, Δ 9-tetrahydrocannabinol, and alcohol, causes ΔFosB to accumulate in addiction-related circuitry (Perrotti et al., 2008), wherein it has been suggested to regulate the expression of several genes commonly associated with this disease (Nestler, 2008; Robison and Nestler, 2011). ΔFosB regulates gene expression via formation of the AP-1 complex that is critically involved in regulation of neuronal activity and behavior after long period of withdrawal (Nestler et al., 2001). ΔFosB may be selectively induced in DYN/substance P containing neurons (Moratalla et al., 1996). Expression of several genes, including *PDYN*, may be regulated by ΔFosB. It has been hypothesized that ΔFosB-mediated suppression of DYN expression in Acb may increase the sensitivity for the rewarding effects of drugs of abuse (Zachariou et al., 2006). These effects may oppose CREB-mediated signaling and ΔFosB is thought to be involved in maintaining addiction-related changes in neurophysiology by: (1) enhancing the rewarding and incentive motivational properties of drugs of abuse via its actions in the Acb and (2) producing tolerance to the cognitive-disrupting effects of such drugs via its actions in the PFC. Relatively short-lived CREB-DYN activity and persistent ΔFosB effects might explain some CREB-DYN mediated behaviors (e.g., tolerance, negative affective states, and depression-like behavior) during early stages of alcohol withdrawal. Alternatively, Δ FosB/DYN-mediated effects may be important during later stages of abstinence from alcohol or drug of abuse (Nestler et al., 2001).

Transcriptional models of addiction involving CREB and ΔFosB gained support in pharmacological and genetic experiments with rodents. However, the promoter and enhancer structure of many human and rodent genes that are implicated in addictive behavior (including *PDYN*) are not completely conserved across species. To address the ΔFosB hypothesis in the context of substance dependence in humans, FosB proteins in human brain were characterized by analysis of postmortem specimens, and compared their levels in the OFC and dlPFC, respectively between human controls and alcoholics (Watanabe et al., 2009). These two sub-regions of the larger PFC are important nuclei within addiction neurocircuitry and chronic exposure to alcohol or other drugs of abuse has been shown to result in accumulation of Δ FosB in both structures (Winstanley et al., 2007; Perrotti et al., 2008). In both the dl-PFC and OFC, as well as in the Acb, three forms of FosB were detected, one of which was ΔFosB. The later protein was found to be expressed at very low, barely detectable levels in all three human brain regions. Importantly no differences in ΔFosB levels were evident between alcoholics and control groups. These human results do not support the ΔFosB hypothesis; they indicate that ΔFosB does not accumulate in the OFC and dlPFC of human alcoholics, suggesting that it may not be directly involved in addiction maintenance, at least not in alcohol dependence.

AP-1 may potentially regulate transcription of human prodynorphin gene in the human brain. Analysis of AP-1 constituents in the human brain demonstrated that canonical and noncanonical prodynorphin AP-1-binding element may be targeted by c-Jun and FosB proteins that form the dominant AP-1 complex (Taqi et al., 2011a). No ΔFosB was found in such a complex. Nonetheless, transcription of human *PDYN* may be regulated by AP-1 forming JunD/FosB heterodimers that binds to a noncanonical AP-1-binding element. This element has a polymorphic site with the T allele conferring AP-1 binding. The C allele of this single nucleotide polymorphism (SNP; rs1997794) that destroys this site represents the allele that is associated with alcohol dependence. Thus, a SNP in the promoter of *PDYN* is associated with alcohol-dependence and may impact *PDYN* transcription in human brain. The impact of genetic variations on *PDYN* transcription may be relevant for diverse adaptive responses of this gene to alcohol.

### Epigenetic evidence

Pleasurable and adverse states resulting from the intake of alcohol and addictive drugs can shape individual differences in the vulnerability to addictive disorders. A fundamental question is how these experiences are encoded at a molecular level in a manner that leads to long-term alterations in plasticity that underlie increased risk for substance abuse. Dysregulation of epigenetic mechanisms could lead to silencing or inappropriate expression of specific genes that could contribute to the pathologies observed in those who are alcohol dependent.

Epigenetics is typically defined as the study of heritable changes in gene expression that are not due to changes in DNA sequence (Eccleston et al., 2007). Hence, identical DNA sequences with differential epigenetic regulation could result in differential gene expression. Epigenetic regulation seems to be time and tissue specific and can be quite diverse even within the same tissue or individual. Epigenetic changes represent alterations in gene expression that are self-perpetuating in the absence of the original signal that caused them (Berger et al., 2009). Environmental factors, including alcohol, can modulate gene expression by inducing alterations in epigenetic markers such as DNA methylation and histone modifications. Epigenetic changes have been associated with a range of neurobiological processes including brain development, synaptic plasticity, learning and memory and neuropathologies such as drug addiction (Borrelli et al., 2008; Renthal and Nestler, 2008; Roth and Sweatt, 2009). DNA methylation is the most stable epigenetic mark that is responsive to environmental stimuli. Environmental conditions can evoke changes in DNA methylation underlying epigenetic re-programming of genes involved in the regulation of addictive disorders. Effects of genetic variations including single nucleotide polymorphisms (SNPs) on DNA methylation may depend on DNA context (Kerkel et al., 2008; Xie et al., 2009; Hellman and Chess, 2010; Shoemaker et al., 2010; Zhang et al., 2010). Importantly, polymorphic positions are most abundant at the CpG dinucleotides that are targets for DNA methylation (Tomso and Bell, 2003; Kerkel et al., 2008; Xie et al., 2009; Hellman and Chess, 2010; Shoemaker et al., 2010; Zhang et al., 2010).

**Figure 3:**
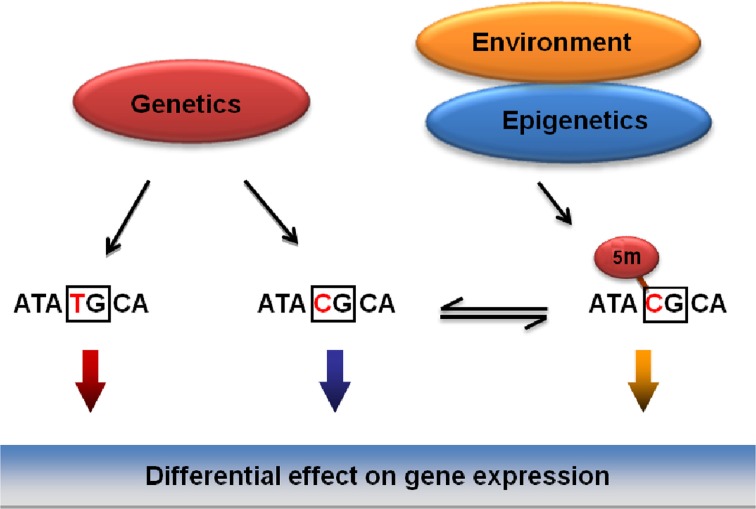
**General model for the integration of genetic, epigenetic and environmental factors on vulnerability to develop addictive disorders.** Mechanistically, the effects of these factors may be integrated through methylation of CpGs overlapping with SNPs (mSNPs) associated with a disease. In the CpG context, the C, one of the two genetic alleles may be methylated and function as two epialleles, the C and ^5^Me-C, that may differentially affect gene expression.

The Bakalkin group has recently addressed the specific hypothesis that genetic, epigenetic and environmental factors associated with a risk for addictive disorders mechanistically converge on SNPs that (1) are associated with addiction, and (2) that overlap with CpG sites thus representing methylation-associated SNPs, or mSNPs (Figure 3) (Taqi et al., 2011b). The two epialleles formed by the unmethylated and methylated C allele at such mSNPs may differentially contribute to disease predisposition, as they may be targeted by transcription factors or by insulator proteins that control the interactions among genomic regulatory elements (for insulators, see (Wallace and Felsenfeld, 2007). This hypothesis (1) unifies the genetic and epigenetic views on the vulnerability to develop addictive disorders, and (2) may explain a part of “missing heritability” not detected in the genome-wide association studies (GWAS).

Our analysis of *PDYN* regulation in the brains of deceased human alcoholics (Taqi et al., 2011b) demonstrated that three *PDYN* SNPs significantly associated with alcohol dependence form CpG sites, and that methylation of one of them was increased and positively correlated with DYN in alcoholics. The mSNP hypothesis has also received support in several recent studies (John et al., 2011; Kaminsky et al., 2012; Martin-Trujillo et al., 2011; Reynard et al., 2011; Ursini et al., 2011). Thus, under influences of the environment—heavy alcohol drinking-induced alterations in methylation of this SNP may affect *PDYN* transcription and, consequently, the vulnerability to develop alcohol dependence. These alterations were observed in brain regions involved in cognitive control of decision-making and may represent molecular adaptations that developed after many years of alcohol exposure and withdrawal. DYN may have a role in the regulation of executive and intellectual functions, learning and memory, and emotions in alcoholics; therefore, their elevation may impair these cognitive processes. Taken together, the results suggest that epigenetic plasticity in the DYN/KOR system may be involved in mediating some of the behavioral effects produced following chronic alcohol exposure.

## Conclusion

The goal of this review is to discuss alcohol-induced plasticity in the DYN/KOR system and how these neuroadaptations may contribute to the pathophysiology of alcohol dependence. The DYN/KOR system has been implicated as an endogenous anti-reward system. However, an upregulated DYN/KOR system in various key brain regions at proximal (DYN/KOR mRNA and expression) and intermediate (CREB/ΔFosB/BDNF mediated signaling) levels could contribute to altered distal events (escalated alcohol use, affective/anxiety like behaviors, sensitization following abstinence) in alcohol dependence. Therefore, increased DYN/KOR activity may induce a negative affective state in withdrawal and provide a basis for the negative reinforcing effects of alcohol. DYN/KORs in the Acb, Amyg and PFC/OFC may mediate the effects of chronic alcohol exposure in a brain region specific manner to decrease positive hedonic states, increase negative affective states or impair decision making and cognitive control, respectively. In addition, epigenetic mechanisms may also be involved in the upregulation of the DYN/KOR system following chronic alcohol exposure. Recent data supporting the role of the DYN/KOR system in mediating negative affective states also help to explain significant co-morbidity between alcohol use disorders and affective disorders. Taken together, the DYN/KOR system is heavily dysregulated in alcohol dependence and represents a potential therapeutic target to combat alcoholism.

### Conflict of interest statement

The authors declare that the research was conducted in the absence of any commercial or financial relationships that could be construed as a potential conflict of interest.
